# Absence of Entourage: Terpenoids Commonly Found in *Cannabis sativa* Do Not Modulate the Functional Activity of Δ^9^-THC at Human CB_1_ and CB_2_ Receptors

**DOI:** 10.1089/can.2019.0016

**Published:** 2019-09-23

**Authors:** Marina Santiago, Shivani Sachdev, Jonathon C. Arnold, Iain S. McGregor, Mark Connor

**Affiliations:** ^1^ Department of Biomedical Sciences, Macquarie University, Sydney, New South Wales, Australia.; ^2^ The Lambert Initiative for Cannabinoid Therapeutics, The University of Sydney, Sydney, New South Wales, Australia.; ^3^ Discipline of Pharmacology, The University of Sydney, Sydney, New South Wales, Australia.; ^4^ School of Psychology, The University of Sydney, Sydney, New South Wales, Australia.

**Keywords:** phytocannabinoid, cannabinoid receptor, terpenoid, entourage effect, THC, signaling

## Abstract

**Introduction:** Compounds present in *Cannabis sativa* such as phytocannabinoids and terpenoids may act in concert to elicit therapeutic effects. Cannabinoids such as Δ^9^-tetrahydrocannabinol (Δ^9^-THC) directly activate cannabinoid receptor 1 (CB_1_) and cannabinoid receptor 2 (CB_2_); however, it is not known if terpenoids present in *Cannabis* also affect cannabinoid receptor signaling. Therefore, we examined six common terpenoids alone, and in combination with cannabinoid receptor agonists, on CB_1_ and CB_2_ signaling *in vitro*.

**Materials and Methods:** Potassium channel activity in AtT20 FlpIn cells transfected with human CB_1_ or CB_2_ receptors was measured in real time using FLIPR^®^ membrane potential dye in a FlexStation 3 plate reader. Terpenoids were tested individually and in combination for periods up to 30 min. Endogenous somatostatin receptors served as a control for direct effects of drugs on potassium channels.

**Results:** α-Pinene, β-pinene, β-caryophyllene, linalool, limonene, and β-myrcene (up to 30–100 μM) did not change membrane potential in AtT20 cells expressing CB_1_ or CB_2_, or affect the response to a maximally effective concentration of the synthetic cannabinoid CP55,940. The presence of individual or a combination of terpenoids did not affect the hyperpolarization produced by Δ^9^-THC (10 μM): (CB_1_: control, 59%±7%; with terpenoids (10 μM each) 55%±4%; CB_2_: Δ^9^-THC 16%±5%, with terpenoids (10 μM each) 17%±4%). To investigate possible effect on desensitization of CB_1_ responses, all six terpenoids were added together with Δ^9^-THC and signaling measured continuously over 30 min. Terpenoids did not affect desensitization, after 30 min the control hyperpolarization recovered by 63%±6% in the presence of the terpenoids recovery was 61%±5%.

**Discussion:** None of the six of the most common terpenoids in *Cannabis* directly activated CB_1_ or CB_2_, or modulated the signaling of the phytocannabinoid agonist Δ^9^-THC. These results suggest that if a phytocannabinoid–terpenoid entourage effect exists, it is not at the CB_1_ or CB_2_ receptor level. It remains possible that terpenoids activate CB_1_ and CB_2_ signaling pathways that do not involve potassium channels; however, it seems more likely that they may act at different molecular target(s) in the neuronal circuits important for the behavioral effect of *Cannabis*.

## Introduction

An enduring notion in the medicinal *Cannabis* and cannabinoid field is that of entourage: the idea that use of the whole plant may exert substantially greater effects than the sum of its individual parts.^1^ Entourage is usually construed as a positive attribute, with the assumption that superior therapeutic actions, or a more favorable “high,” will be obtained from consuming the whole *Cannabis* plant rather than individual components such as Δ^9^-tetrahydrocannabinol (Δ^9^-THC). Somewhat surprisingly, the evidence for this widely cited notion is relatively sparse.

*Cannabis* contains ∼150 phytocannabinoids, the most common of which are Δ^9^-THC and cannabidiol (CBD), together with their acid precursors THCA and CBDA.^2^ *Cannabis* also contains a large number of monoterpene and sesquiterpene compounds (together called terpenoids), the most common of which include α-pinene, β-pinene, linalool, limonene and β-myrcene (monoterpenes) and β-caryophyllene and caryophyllene oxide (sesquiterpenes).^3^ Terpenoids are volatile compounds that are synthesized alongside phytocannabinoids mainly in the trichomes of the cannabis plant, and provide cannabis with its distinctive aroma and flavor.^4^ Terpenoids are often lost if the extraction process involves heating.^5^

The entourage concept applied to cannabis can encompass the potential for both cannabinoid–cannabinoid and cannabinoid–terpenoid interactions. With regard to the former, Δ^9^-THC-CBD synergy in producing analgesia was reported in an animal model of neuropathic pain^6^ while in humans, CBD has been proposed to ameliorate some of the adverse psychotomimetic and anxiogenic effects of Δ^9^-THC.^7,8^ This claim is controversial, however, with a number of contrary findings.^9,10^ CBD may modulate Δ^9^-THC effects at the receptor level acting as a CB_1_ negative allosteric modulator,^11^ providing some biological plausibility to a modulatory interaction.

Scientific evidence for cannabinoid–terpenoid interactions is essentially absent, and mostly comes from websites and dispensaries extolling the virtues of proprietary *Cannabis* chemical varieties, or chemovars.^12,13^ However, some terpenoids do have intrinsic psychoactive and physiological effects, and modulatory effects on Δ^9^-THC actions are not farfetched.^1,14^ For example, in studies with laboratory animals, limonene displayed anxiolytic effects, pinene increased gastrointestinal motility, linalool was sedative, anticonvulsant, and anxiolytic, while myrcene produced sedation, analgesia, and muscle relaxant effects (summarized in Russo and Marcu^14^). Lewis et al.^13^ reported that in a low terpenoids variety (1.1% terpenoids) myrcene concentration is 0.45%, while in a high variety (4.8% total) myrcene concentration is as high as 3.44%. Compelling evidence for cannabinoid–terpenoid interactions or synergy does not yet exist. A report on perceived efficacy of Cannabis for childhood epilepsy identified the presence of three predominant terpenoids (β-caryophyllene, β-myrcene, and α-pinene); however, when extracts perceived as “effective” were compared with “ineffective” extracts, differences in terpenoid profile/content were not significant.^15^

With so many bioactive components present in cannabis, the systematic, granular elucidation of possible entourage effects poses a substantial combinatorial puzzle and scientific challenge. As a preliminary approach to addressing this challenge, this study examined whether the effects of Δ^9^-THC on its cognate cannabinoid receptors (CB_1_ and CB_2_) would be modified in the presence of terpenoids that are commonly found in cannabis, either alone or in combination. The demonstration of such a receptor-level entourage effect might lead to predictions regarding functional cannabinoid–terpenoid interaction effects that could be tested *in vivo*.

## Materials and Methods

### Cell culture

Experiments used mouse wild-type AtT20 FlpIn cells (AtT20-WT), or these cells stably transfected with human CB_1_ or CB_2_ receptors with 3×N-terminus hemagglutinin tags (AtT20-CB_1_ and AtT20-CB_2_, respectively).^16^ Cells were cultivated in Dulbecco's modified Eagle's medium (DMEM; Sigma-Aldrich) supplemented with 10% fetal bovine serum (FBS; Sigma/SAFC) and 100 U penicillin/100 μg streptomycin mL^−1^ (Gibco). Selection antibiotics were 80 μg mL^−1^ Zeocin (Invivogen) for AtT20-WT or 80 μg mL^−1^ hygromycin B Gold (Invivogen) for transfected cells.

Cells were grown in 75 mm^2^ flasks at 37°C/5% CO_2_ and passaged when 80–90% confluent. Assays were carried out on cells up to 20 passages in culture.

### Potassium channel activity measurements

Changes in membrane potential were measured using the FLIPR^®^ blue membrane potential dye (Molecular Devices) in a FlexStation 3, as outlined in Knapman 2013.^17^ Cells from a 90–100% confluent 75 mm^2^ flask were resuspended in Leibovitz's L-15 Medium (Gibco) supplemented with 1% FBS, 100 U penicillin/100 μg streptomycin mL^−1^, and glucose (15 mM) and plated in 96-well black-walled clear bottom microplates (Costar) in a volume of 90 μL per well. Cells were incubated overnight in humidified ambient air at 37°C incubator. Membrane potential dye, used at 50% of the manufacturer-recommended concentration, was resuspended in Hank's Balanced Salt Solution (HBSS) of composition (in mM): NaCl 145, HEPES 22, Na_2_HPO_4_ 0.338, NaHCO_3_ 4.17, KH_2_PO_4_ 0.441, MgSO_4_ 0.407, MgCl_2_ 0.493, CaCl_2_ 1.26, glucose 5.55 (pH 7.4, osmolarity 315±15). Dye was loaded onto each well (90 μL per well) and equilibrated at 37°C for ∼1 h before assay. Fluorescence was measured every 2 sec (*λ* excitation=530 nm, *λ* emission=565 nm, *λ* emission cutoff=550 nm). Assays were carried out at 37°C, and drugs were automatically added in volumes of 20 μL.

#### Determining the effects of terpenoids on acute hyperpolarization

Terpenoids were added after ∼60 sec of baseline recording and incubated for 5 min before cannabinoid (CP55,940 or Δ^9^-THC) addition. In AtT20-WT cells, somatostatin (SST) was added instead of cannabinoid.

#### Determining the effects of terpenoids on signaling desensitization

Homologous desensitization was measured by simultaneously adding Δ^9^-THC with terpenoids after 120 sec of baseline recording. Signaling desensitization was calculated as percentage decrease from peak Δ^9^-THC response after 30 min in drugs. SST (100 nM) was added 30 min after Δ^9^-THC addition to examine the potential effects of prolonged cannabinoid receptor activation on native SST receptors (heterologous desensitization). The SST response was compared between groups (with or without terpenoids).

#### Drug dilution

All drugs (except SST) were prepared in dimethyl sulfoxide (DMSO) and stored as frozen stocks at a concentration of 10–100 mM. Terpenoid stock solution concentrations were 100 mM, with the exception of β-myrcene (30 mM), which was insoluble at 100 mM. SST was dissolved in water. Fresh aliquots were used each day, with the drugs diluted in HBSS containing 0.1% bovine serum albumin (Sigma-Aldrich) immediately before the assay. The final concentration of DMSO in each well was 0.1–0.11%; this limited the maximum concentration of terpenoids able to be tested. A vehicle (HBSS plus solvent alone) well was included in each column of the 96-well plate, and the changes in fluorescence produced by vehicle alone were subtracted before determining the maximum hyperpolarization after each drug exposure.

### Drugs and reagents

Δ^9^-THC was obtained from THCPharm (Frankfurt, Germany). Terpenoids were obtained from Sigma-Aldrich; (+)-α-pinene, (+)-β-pinene, (−)-β-caryophyllene, (+/−)-linalool, (R)-(+)-limonene, and β-myrcene. SST was obtained from Auspep and CP55,940 from Cayman. Unless otherwise indicated, the other chemicals and reagents were obtained from Sigma-Aldrich.

### Data analysis

Each experiment was independently repeated at least five times, with two technical replicates in each determination. Data are expressed as a percentage change in the fluorescence compared with the predrug baseline (30 sec before drug addition) or as a percentage of 1 μM CP55,940 response. Graphs were plotted using Graphpad Prism 7.02, and scatter dot plots show means with standard error of the mean. Means were compared using unpaired Student's *t*-test or no matching one-way analysis of variance, followed by correction for multiple comparisons (Dunnett); and null hypothesis was rejected if *p*-value was <0.05 (*p*>0.05=not significant).

## Results

### Terpenoids in AtT20-WT cells

**FIG. 1.**
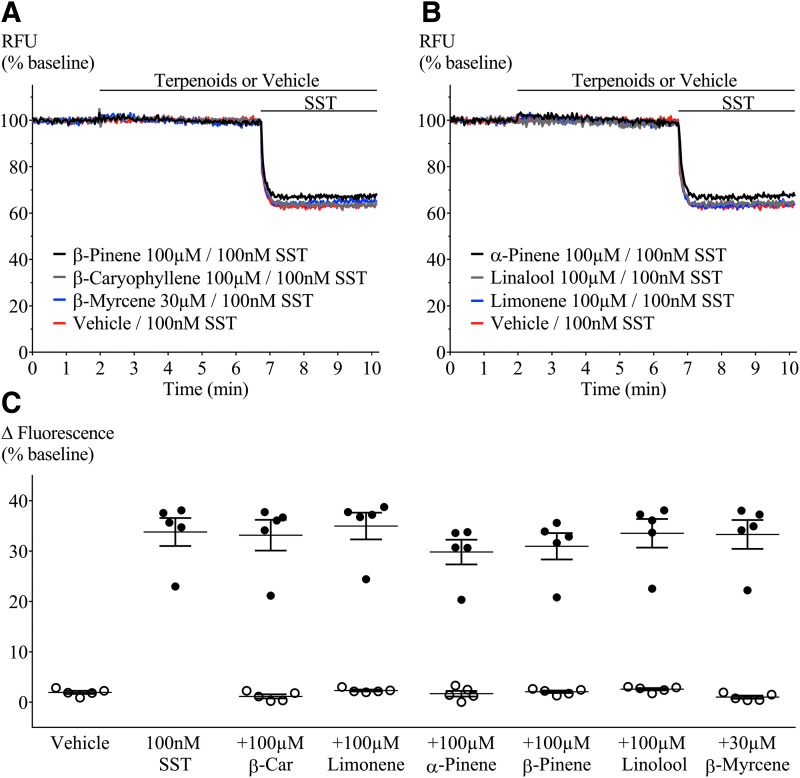
Terpenoid- and SST-mediated fluorescence change in AtT20-WT. Representative traces showing change in fluorescence signal after terpenoid and SST (100 nM) application. A decrease in signal corresponds to membrane hyperpolarization. Addition of terpenoids **(A)** β-pinene, β-caryophyllene, and β-myrcene; **(B)** α-pinene, linalool, and limonene did not change baseline fluorescence, while SST mediated a clear hyperpolarization. **(C)** Percentage change of fluorescence from baseline after each terpenoid (open circles) and SST (closed circles) application. Terpenoids were added at 2 min; 5 min before SST. When compared with positive (SST) or negative (vehicle) controls, none of the terpenoids tested affected baseline membrane potential or peak SST response. β-Car=β-caryophyllene. *n*=5, SEM, one-way ANOVA *p*>0.05. Drugs were added for the duration of the bar. ANOVA, analysis of variance; SEM, standard error of the mean; SST, somatostatin.

We first examined terpenoid action in nontransfected AtT20 cells. We used SST (100 nM) as a positive control because it hyperpolarizes AtT20-WT cells through activation of endogenous SST receptors (Fig. 1A, B).^17,18^ Addition of α-pinene, β-pinene, β-caryophyllene, linalool, limonene (100 μM), or β-myrcene (30 μM) did not affect the membrane potential of AtT20-WT cells (Fig. 1C, open circles). The presence of terpenoids (100 μM/30 μM) had no effect on the subsequent SST response (Fig. 1C).

### Terpenoids in AtT20-CB_1_ and -CB_2_ cells

**FIG. 2.**
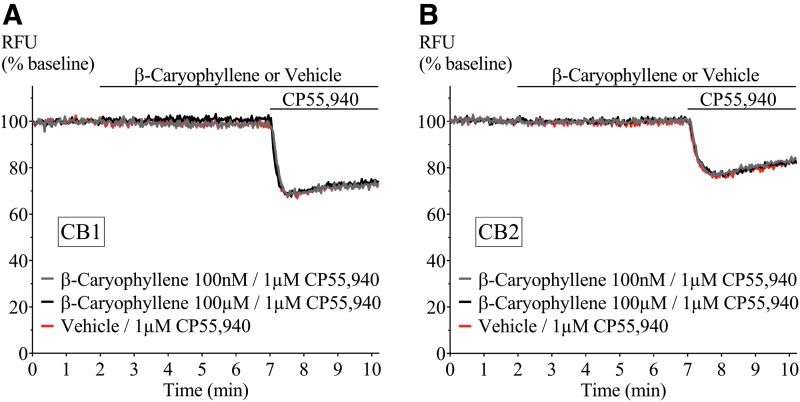
Representative traces of β-caryophyllene and CP55,940 in AtT20-CB_1_ and -CB_2_. Fluorescence was recorded for 10 min where β-caryophyllene (100 nM and 100 μM) was added at 2 min followed by incubation for 5 min, before 1 μM CP55,940 application. β-caryophyllene did not hyperpolarize **(A)** AtT20-CB_1_ and **(B)** AtT20-CB_2_ cells, or affect the response to CP55,940 (1 μM). Drugs were added for the duration of the bar. CB_1_, cannabinoid receptor 1, CB_2_, cannabinoid receptor 2.

**FIG. 3.**
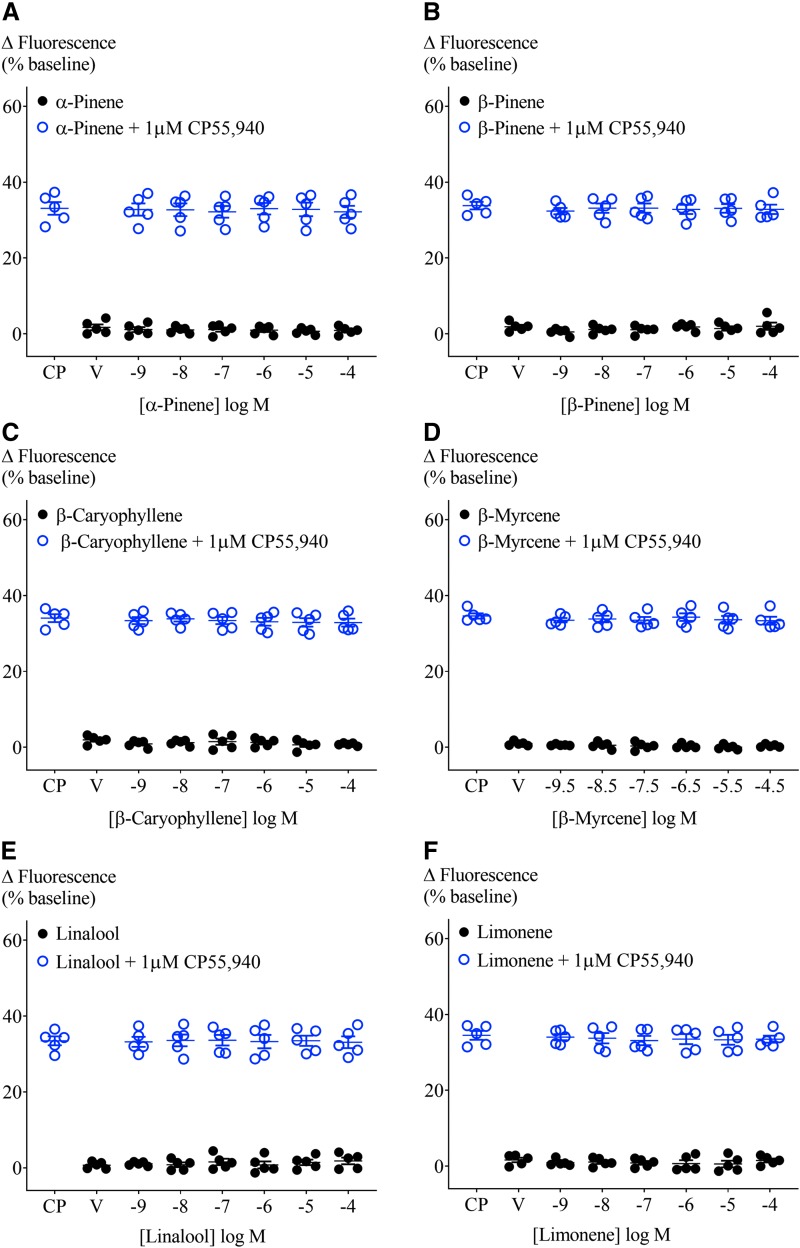
Effect of terpenoids at varying concentrations on AtT20-CB_1_ membrane potential and on 1 μM CP55,940-induced hyperpolarization. Terpenoids **(A)** α-pinene, **(B)** β-pinene, **(C)** β-caryophyllene, **(D)** β-myrcene, **(E)** linalool, and **(F)** limonene were added to AtT20-CB_1_ cells and incubated for 5 min. Maximum fluorescence changes were not different from negative control (closed circles, *n*=5, SEM, one-way ANOVA *p*>0.05). CP55,940 (1 μM) addition to AtT20-CB_1_ cells induced fluorescence changes from 33.1%±1.7% to 34.6%±0.7%. Peak CP55,940 responses were not affected by the presence of terpenoids (open circles, *n*=5, SEM, one-way ANOVA *p*>0.05). V, vehicle.

The absence of a terpenoid response in AtT20-WT cells enabled the study of their effect on membrane potential in AtT20 cells expressing human CB_1_ or CB_2_. We examined whether terpenoids (1 nM–100 μM, β-myrcene 300 pM–30 μM) hyperpolarized cells through these receptors and, in parallel, whether they affected a subsequent response to a maximally effective concentration of CP55,940 (1 μM; Fig. 2).^16^ A summary of the fluorescence change after terpenoid addition to AtT20-CB_1_ cells is shown in Figure 3 (closed circles). No difference between vehicle and terpenoids was observed. Further, none of the terpenoids changed the membrane potential of cells expressing CB_2_ (Supplementary Fig. S1). The change in fluorescence produced by the subsequent addition of the nonselective cannabinoid agonist CP55,940 (1 μM) was also unaffected in both AtT20-CB_1_ and -CB_2_ (Fig. 3 and Supplementary Fig. S1, open circles).

**FIG. 4.**
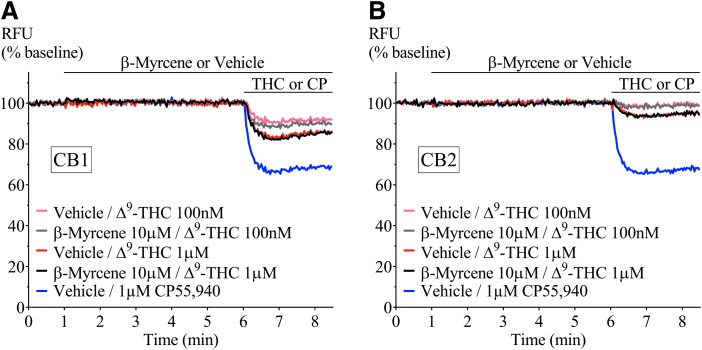
Representative traces of β-myrcene and Δ^9^-THC in **(A)** AtT20-CB_1_ and **(B)** AtT20-CB_2_. Fluorescence change mediated by two submaximal concentrations of Δ^9^-THC (100 nM and 1 μM) in the presence of β-myrcene (10 μM). Terpenoid was added at 1 min and incubated for 5 min before Δ^9^-THC application. CP55,940 added as positive control. Drugs were added for the duration of the bar. Δ^9^-THC, Δ9-tetrahydrocannabinol.

**FIG. 5.**
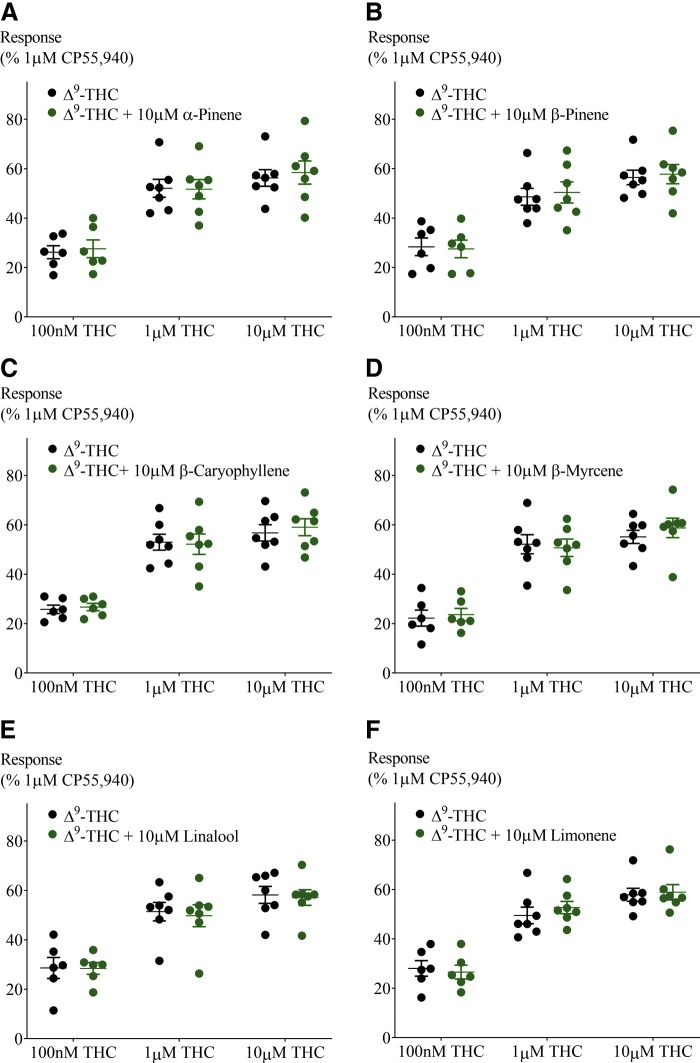
Effect of 10 μM terpenoids on Δ^9^-THC-induced hyperpolarization in AtT20-CB_1_. Terpenoids tested were **(A)** α-pinene, **(B)** β-pinene, **(C)** β-caryophyllene, **(D)** β-myrcene, **(E)** linalool, and **(F)** limonene. Response to Δ^9^-THC at two submaximal and one maximal concentration (*n*=6–7, SEM, unpaired *t*-test *p*>0.13). Data presented as % of maximum CP55,940 (1 μM) response.

**FIG. 6.**
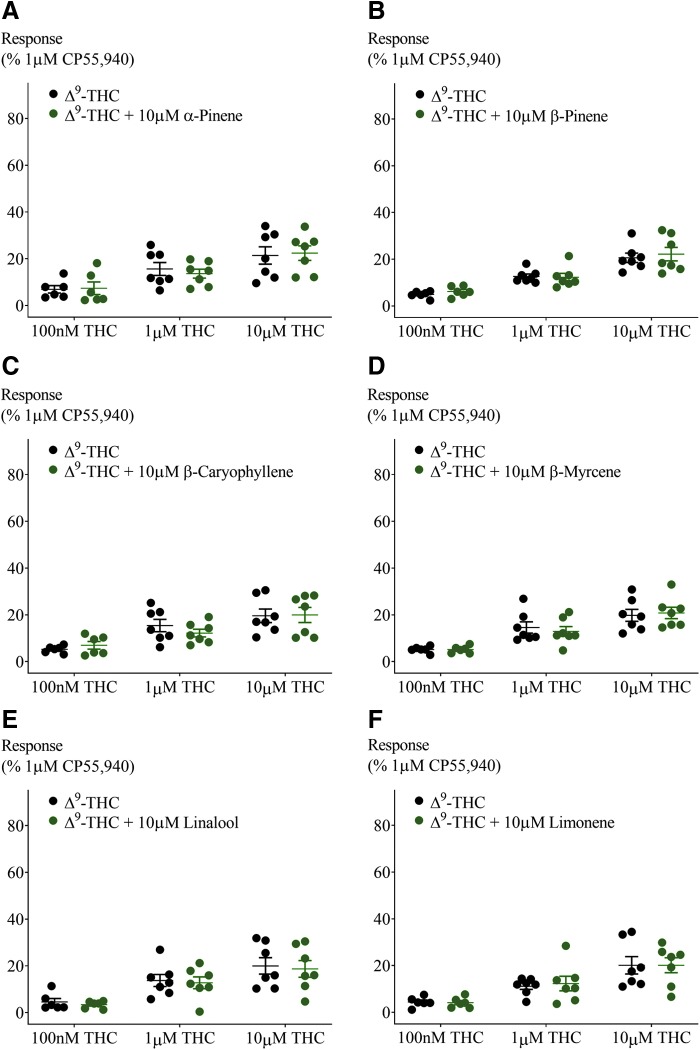
Effect of 10 μM terpenoids on Δ^9^-THC-induced hyperpolarization in AtT20-CB_2_. Terpenoids tested were **(A)** α-pinene, **(B)** β-pinene, **(C)** β-caryophyllene, **(D)** β-myrcene, **(E)** linalool, and **(F)** limonene. Response to Δ^9^-THC at two submaximal and one maximal concentration (*n*=6–7, SEM, unpaired *t*-test *p*>0.26). Data presented as % of maximum CP55,940 (1 μM) response.

**FIG. 7.**
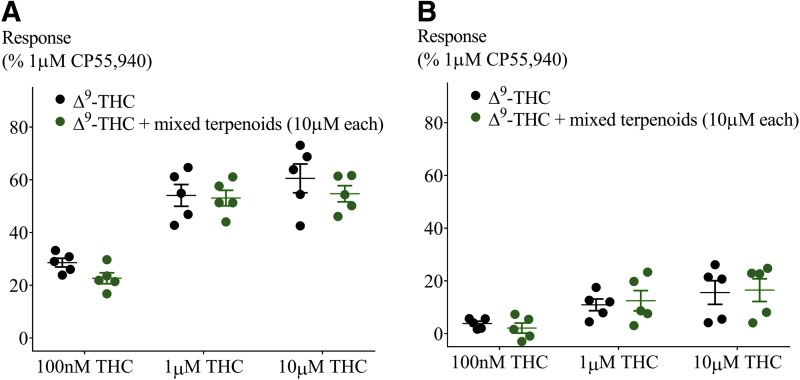
Testing the “Entourage effect.” Effect of combination of six terpenoids at 10 μM each on Δ^9^-THC-induced hyperpolarization in **(A)** AtT20-CB_1_ and **(B)** AtT20-CB_2_. Response to Δ^9^-THC at two submaximal and one maximal concentration (*n*=5, SEM, unpaired *t*-test *p*>0.13). Data presented as % of maximum CP55,940 (1 μM) response.

CP55,940 is a high-efficacy agonist of both CB_1_ and CB_2_ receptors.^19^ However, in *Cannabis*, Δ^9^-THC is the principle cannabinoid agonist, and it has a lower efficacy than CP55,940, which is apparent in the hyperpolarization assay as a lower maximal response.^19^ We next tested the effect of a low and high concentration of terpenoids (100 nM and 10 μM) on the hyperpolarization produced by three concentrations of Δ^9^-THC (100 nM, 1 and 10 μM). Application of Δ^9^-THC, after 5 min of individual terpenoid application, produced a fluorescence change (Fig. 4) that was not significantly different from that produced by Δ^9^-THC alone in both AtT20-CB_1_ and -CB_2_ cells (10 μM Δ^9^-THC, Figs. 5 and 6; 100 nM Δ^9^-THC, Supplementary Figs. S2 and S3). To explore the possibility of an emergent entourage effect, we combined all six terpenoids (10 μM each) and tested the effect of the mixture on the Δ^9^-THC-induced hyperpolarization. Similar to individually tested terpenoids, the effects of Δ^9^-THC were not changed by the mixture (Fig. 7).

### Terpenoids and desensitization in AtT20-CB_1_

**FIG. 8.**
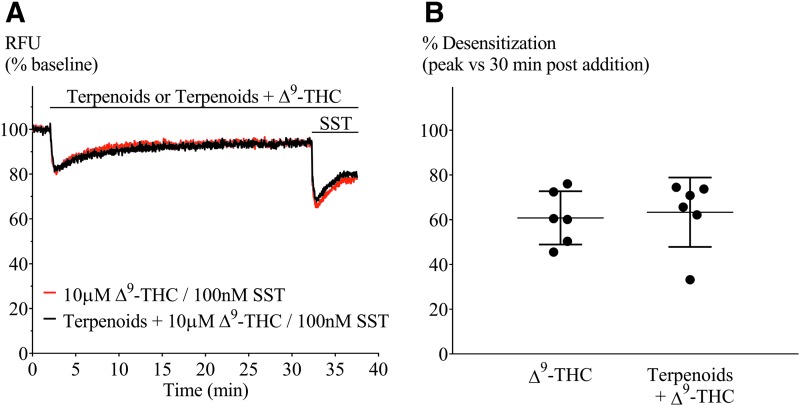
Terpenoids on Δ^9^-THC-mediated desensitization in AtT20-CB_1_. **(A)** Representative traces of hyperpolarization and signal desensitization mediated by Δ^9^-THC alone (10 μM, black) or with terpenoids (10 μM each, red). Cells were then challenged with SST (100 nM) after 30 min to examine heterologous desensitization. **(B)** Percentage desensitization after 30 min exposure to Δ^9^-THC alone (10 μM) or in the presence of terpenoids (10 μM each), compared with peak fluorescence response. Terpenoids did not affect Δ^9^-THC-mediated desensitization (*n*=5, SEM, unpaired *t*-test *p*=0.76). Drugs were added for the duration of the bar.

**FIG. 9.**
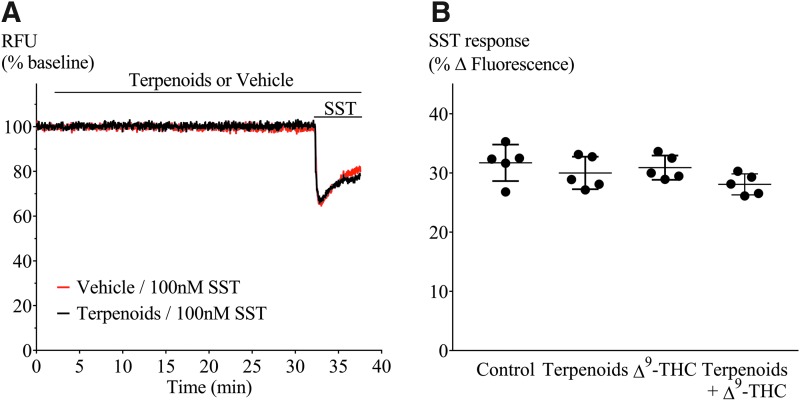
SST challenge of AtT20-CB_1_ cells to investigate heterologous desensitization. **(A)** Representative traces of cells preincubated with (black) or without (red) terpenoids for 30 min before SST (100 nM) challenge. **(B)** Comparison of peak hyperpolarization (% fluorescence change) obtained after SST (100 nM) challenge (*n*=5, one-way ANOVA *p*>0.05). Drugs were added for the duration of the bar.

We have previously reported desensitization cannabinoid-mediated cellular hyperpolarization in AtT20 cells expressing rat or human CB_1_ receptors,^20,21^ and we found that this reversal of CP55,940-induced hyperpolarization was accelerated by negative allosteric modulators such as ORG27569 and PSNCBAM-1. Therefore, we tested whether terpenoids may act in a similar way to ORG27569 and other negative allosteric modulators, altering desensitization time course. We used Δ^9^-THC instead of CP55,940, as Δ^9^-THC is the main phytocannabinoid agonist. Prolonged application of Δ^9^-THC (10 μM) produced a hyperpolarization that reversed substantially over 30 min. Representative traces for this experiment are illustrated in Figure 8A. We measured the peak response to Δ^9^-THC and the signal remaining 30 min after agonist exposure, and quantified desensitization as a percentage decline in the peak response. The Δ^9^-THC (10 μM) signal desensitized by 63%±6%, in the presence of the terpenoid mix desensitization, was 61%±5% (Fig. 8B). Thus, terpenoids did not interfere with desensitization of CB_1_ signaling produced by Δ^9^-THC. We also assessed the capacity of Δ^9^-THC alone, terpenoids alone (10 μM each), or terpenoids combined with Δ^9^-THC to affect SST receptor signaling in AtT20-CB_1_ cells (heterologous desensitization). SST (100 nM) was applied 30 min after first drug application (Figs. 8A and 9A), and the hyperpolarization produced by SST after Δ^9^-THC, terpenoids alone, or Δ^9^-THC with terpenoids was not significantly different from that produced by SST alone (*p*>0.05, Fig. 9B).

## Discussion

The principal finding of this study is that agonist activation of CB_1_ and CB_2_ receptors is not obviously altered by any or all of the six major terpenoids from *Cannabis sativa*. The terpenoids tested did not activate CB_1_ or CB_2_ by themselves, nor did they modify the signaling of the high-efficacy agonist CP55,940 or the lower efficacy agonist Δ^9^-THC. In particular, Δ^9^-THC effects would be expected to be very sensitive to the presence of drugs that inhibited (or enhanced) signaling at the receptor. There are no spare receptors for Δ^9^-THC in this assay, and changes in ligand binding would be directly reflected as a change in the maximum response. The lack of effect of terpenoids on the response to the synthetic cannabinoid CP55,940 indicates that terpenoids do not interfere with maximal cannabinoid receptor-mediated hyperpolarization, suggesting no direct modulation of the potassium channel response. This was confirmed by the lack of effect of terpenoids on the response to SST.

A previous study showed that β-caryophyllene is a CB_2_ agonist.^22^ However, we were unable to detect any effect of β-caryophyllene on CB_2_ signaling in this study. The reasons for this are unclear, but the efficacy of β-caryophyllene has not been defined in cellular assays and may be lower than that of Δ^9^-THC. The CB_2_ response to even high concentrations of Δ^9^-THC in our assay is small, suggesting that productive coupling of CB_2_ to endogenous potassium channels in AtT20 cells requires high-efficacy agonists. The affinity of β-caryophyllene for CB_2_ (155 nM) has been determined in membranes from HEK293 cells heterologously expressing CB_2_,^22^ but is not known in intact cells. Its EC_50_ for inhibition of forskolin-induced adenylyl cyclase in CHO-K1 expressing CB_2_ was ∼2 μM,^22^ suggesting a low functional affinity, which may not be sufficient to significantly affect the rapid response to the higher affinity agonist Δ^9^-THC.

The role of terpenoids in cannabis-induced analgesia in rats was recently evaluated by Harris et al.^23^ They tested THC, isolated terpenoids, extract without terpenoids, and full extract, and suggested that the analgesic effect of cannabis is mainly due to THC presence and proposed that terpenoids do not contribute to cannabis-mediated analgesia. These findings support our results, and interestingly their extract had a very high percentage of β-caryophyllene.

Positive and negative allosteric modulators have been reported for CB_1_,^24,25^ and the effects of several negative allosteric modulators have been defined in the hyperpolarization assay used here.^20^ Both PSNCBAM-1 and ORG27569 enhanced CP55,940 signal desensitization, while PSNCBAM-1 also inhibited the initial CP55,940 hyperpolarization. Coapplication of the terpenoids with Δ^9^-THC failed to affect the peak response, or the degree of tachyphylaxis observed over a 30-min exposure to drug, suggesting that they are not acting as allosteric modulators of this CB_1_ signaling pathway.

### Limitations

A limitation of this study is that we only examined CB_1_ and CB_2_ signaling through one pathway, involving Gi/o. The hyperpolarization of the AtT20 cells likely represents G-protein-mediated activation of inwardly rectifying potassium channels (GIRK), as previously described for CB_1_ and other GPCR in these cells as well as in several different neurons.^26–28^ Cannabinoid receptors couple to multiple G proteins as well as signaling through other pathways such as those dependent on arrestins, and it is possible that entourage effects of terpenoids are mediated through modulation of a subset of the cannabinoid receptor signaling repertoire.^26^ CB_1_ and CB_2_ receptors can be activated in a ligand-biased manner—the phenomenon where a drug preferentially activates a subset of the signaling pathways that the receptor can access.^29^ In general, this bias has been best defined for G protein coupling versus activation of arrestin-mediated signaling, but to our knowledge there are no examples of cannabinoid ligands only affecting arrestin-mediated signaling.^19,30^ It remains possible that terpenoids have such an absolute bias, but this would be unprecedented, and in any case recruitment of arrestin would be expected to produce enhanced desensitization of the CB_1_ responses to prolonged agonist exposure.^20,29^ Any subtle change to receptor signaling should be clear with use of the low-efficacy agonist Δ^9^-THC.

Overall, our data suggest that it is unlikely that the terpenoids studied here affect Δ^9^-THC interactions with cannabinoid receptors. However, this is not a definitive rebuttal of the entourage effect. Our study cannot address the possibility of entourage effects emerging through effects of terpenoids on cannabinoid metabolism and distribution as well as interaction with other G-protein-coupled receptors, ligand-gated ion channels, signaling cascades present on the same cells that express cannabinoid receptors, or on other cells up or downstream of the cannabinoid receptor expressing cells. There are many other ways that these molecules could interact with cannabinoids to influence the overall therapeutic and subjective outcomes of cannabis administration, and it should be acknowledged that Δ^9^-THC influences signaling at a wide variety of other noncannabinoid receptor targets (see Banister et al.^31^ for a review). Terpenoids may even have primary effects on distinct functional modules that together with cannabinoid receptor-modulated pathways are ultimately integrated into a behavioral or physiological output. So the quest for entourage does not end here; in many ways, it has only just begun.

## Supplementary Material

Supplemental data

Supplemental data

Supplemental data

## Abbreviations Used

Δ^9^-THC: Δ^9^-tetrahydrocannabinol
β-Car: β-caryophyllene
ANOVA: analysis of variance
CBD: cannabidiol
DMEM: Dulbecco's modified Eagle's medium
DMSO: dimethyl sulfoxide
FBS: fetal bovine serum
HBSS: Hank's Balanced Salt Solution
RFU: relative fluorescence units
SEM: standard error of the mean
SST: somatostatin
V: vehicle
