# Supplementary material for: Absence of Entourage: Terpenoids Commonly Found in Cannabis sativa Do Not Modulate the Functional Activity of Δ9-THC at Human CB1 and CB2 Receptors
Source: Cannabis Cannabinoid Res. 2019 Sep 23;4(3):165–76. doi: 10.1089/can.2019.0016 (PMC6757242; doi:10.1089/can.2019.0016)
Supplement: Supplemental data [file Supp_Fig1.pdf]

Supplementary Data

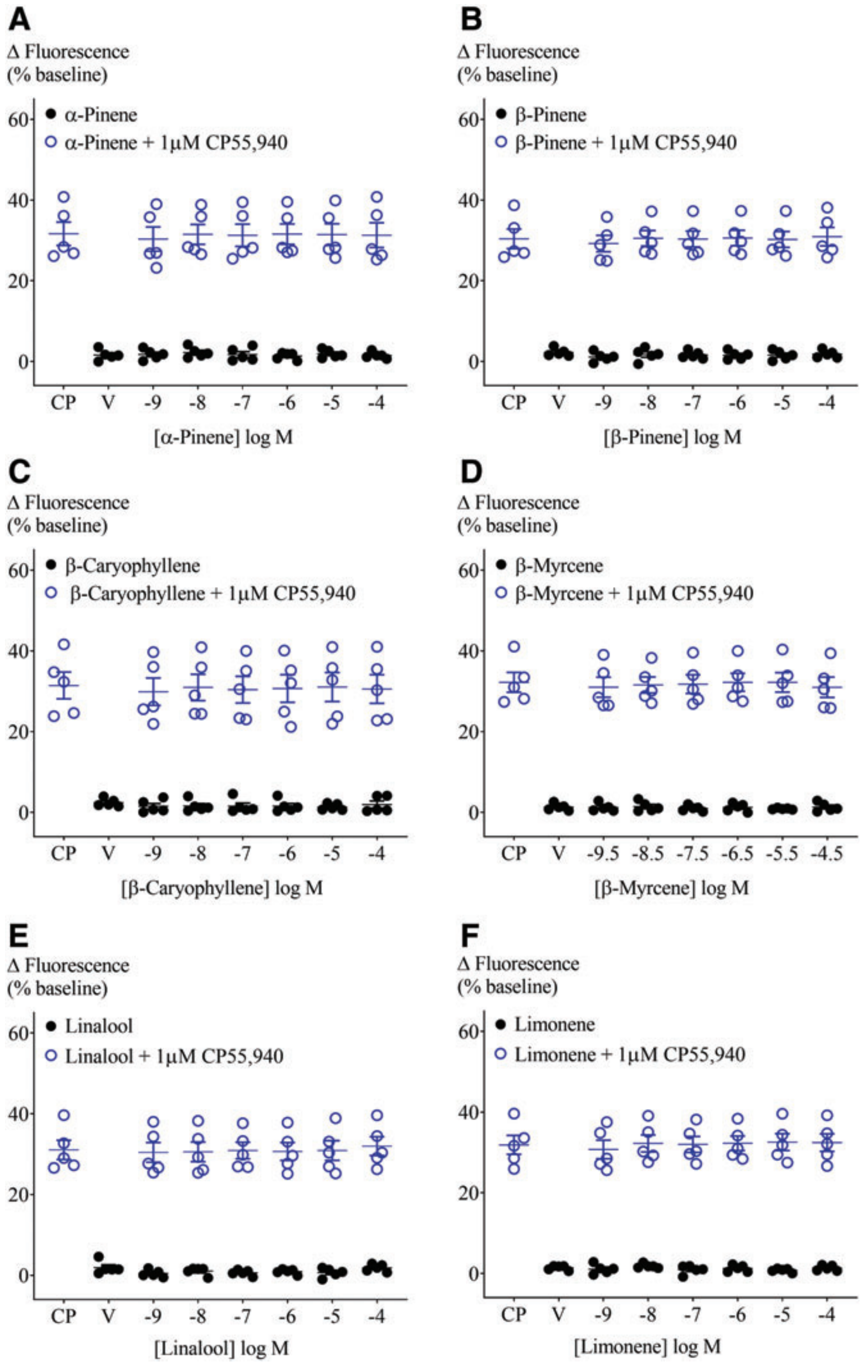

SUPPLEMENTARY FIG. S1. Effect of terpenoids at varying concentrations on AtT20-CB_2 membrane potential and on 1 μM CP55,940-induced hyperpolarization. Terpenoids **(A)** α-pinene, **(B)** β-pinene, **(C)** β-caryophyllene, **(D)** β-myrcene, **(E)** linalool, and **(F)** limonene were added to AtT20-CB_2 cells and incubated for 5 min. Maximum fluorescence changes were determined and compared with negative control (HBSS, closed circles). No significant fluorescence difference was observed when comparing means of terpenoids and HBSS ($n = 5$, SEM, unpaired t-test $p = 0.72$). CP55,940 (1 μM) addition to AtT20-CB_2 cells induced fluorescence changes from 30.4% ± 2.4% to 32.2% ± 2.5%. Peak CP55,940 responses were not affected by the presence of terpenoids (open circles, $n = 5$, SEM, unpaired t-test $p > 0.09$). CB_2, cannabinoid receptor 2; HBSS, Hank's Balanced Salt solution; SEM, standard error of the mean; V, vehicle
