# Supplementary material for: Absence of Entourage: Terpenoids Commonly Found in Cannabis sativa Do Not Modulate the Functional Activity of Δ9-THC at Human CB1 and CB2 Receptors
Source: Cannabis Cannabinoid Res. 2019 Sep 23;4(3):165–76. doi: 10.1089/can.2019.0016 (PMC6757242; doi:10.1089/can.2019.0016)
Supplement: Supplemental data [file Supp_Fig2.pdf]

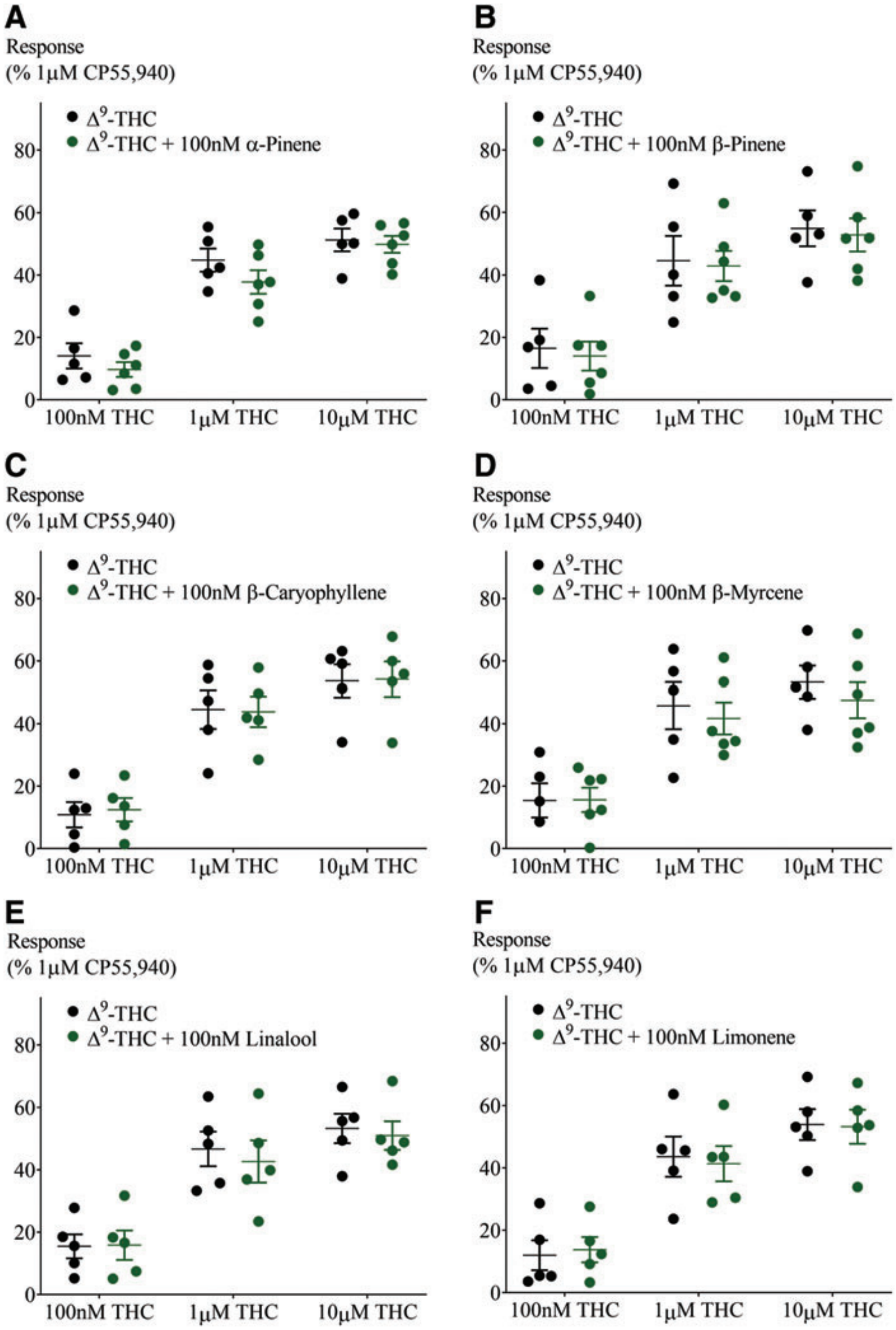

SUPPLEMENTARY FIG. S2. Effect of 100 nM terpenoids on peak hyperpolarization induced by Δ^9-THC in AtT20-CB_1 cells. Terpenoids tested were **(A)** α-pinene, **(B)** β-pinene, **(C)** β-caryophyllene, **(D)** β-myrcene, **(E)** linalool, and **(F)** limonene. Response to Δ^9-THC at two submaximal and one maximal concentration ($n = 5$, SEM, unpaired t-test $p > 0.24$). Data presented as % of maximum CP55,940 (1 μM) response. Δ^9-THC, Δ9-tetrahydrocannabinol; CB_1, cannabinoid receptor 1.
